# Alkaloids from *Hippeastrum papilio*

**DOI:** 10.3390/molecules16087097

**Published:** 2011-08-18

**Authors:** Jean Paulo de Andrade, Strahil Berkov, Francesc Viladomat, Carles Codina, José Angelo S. Zuanazzi, Jaume Bastida

**Affiliations:** 1 Departament de Products Naturals, Biologia Vegetal i Edafologia, Facultat de Farmàcia, Universitat de Barcelona, Av. Joan XXIII s/n, E-08028 Barcelona, Spain; 2 Faculdade de Farmácia, Universidade Federal do Rio Grande do Sul, Av. Ipiranga 2752, 90610-000, Porto Alegre, RS, Brazil; 3 AgroBioInstitute, 8 Dragan Tzankov Blvd., 1164 Sofia, Bulgaria

**Keywords:** galanthamine, 11*β*-hydroxygalanthamine, *Hippeastrum papilio*, 2D NMR

## Abstract

Galanthamine, an acetylcholinesterase inhibitor marketed as a hydrobromide salt (Razadyne®, Reminyl®) for the treatment of Alzheimer’s disease (AD), is obtained from Amaryllidaceae plants, especially those belonging to the genera *Leucojum*, *Narcissus*, *Lycoris* and *Ungernia*. The growing demand for galanthamine has prompted searches for new sources of this compound, as well as other bioactive alkaloids for the treatment of AD. In this paper we report the isolation of the new alkaloid 11*β*-hydroxygalanthamine, an epimer of the previously isolated alkaloid habranthine, which was identified using NMR techniques. It has been shown that 11*β*-hydroxygalanthamine has an important *in vitro* acetylcholinesterase inhibitory activity. Additionally, *Hippeastrum papilio* yielded substantial quantities of galanthamine.

## 1. Introduction

The object of many studies, Amaryllidaceae alkaloids contain a wide range of chemical structures and interesting biological properties [1], showing pronounced antimalarial [2], antitumoral [3] and acetylcholinesterase inhibitory activity [4]. The use of galanthamine in palliative therapy for mild-moderate AD [5] has prompted the search for analogous compounds bearing the galanthamine-type skeleton. Additionally, as most of the galanthamine used in clinics is supplied from natural sources, there is considerable interest in finding new Amaryllidaceae species for a sustainable production of galanthamine [4,6].

Plants of the genus *Hippeastrum*, which is endemic to South America, have yielded interesting bioactive compounds such as montanine, with significant psychopharmacological and acetylcholinesterase inhibitory activity [7,8] and candimine, active against *Trychomonas vaginalis* [9]. Recent nrDNA ITS sequences data have included it within the Hippeastroid subclade and alluded to a probable Brazilian origin [10].

We have identified six known alkaloids, including significant quantities of galanthamine which was the main alkaloid isolated, from the bulbs and leaves of *Hippeastrum papilio* (Ravenna) Van Scheepen, which grows in Southern Brazil. Furthermore, we have clarified the correct position of the hydroxyl-substituent in the alkaloid habranthine through the isolation of its epimer, the new alkaloid 11*β*-hydroxygalanthamine. Habranthine was isolated from *Pancratium maritimum* without certainty about the stereochemistry of the hydroxyl substituent at position 11 in the galanthamine-type skeleton. Using modern 2D NMR, we report the correct assignment of 11*β*-hydroxygalanthamine, a new alkaloid from *Hippeastrum papilio*, confirming that the previously reported habranthine is in fact 11*α*-hydroxygalanthamine. Furthermore, 11*β*-hydroxygalathamine has demonstrated a good ability to inhibit the acetylcholinesterase enzyme, with IC_50_ of 14.5 ± 0.33 µM.

## 2. Results and Discussion

**Figure 1:**
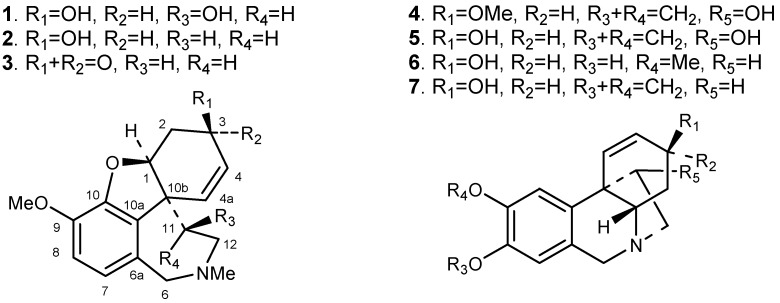
Isolated alkaloids from *Hippeastrum papilio*.

Bulbs and leaves of the plant showed similar alkaloid profiles by analytical TLC. After Vaccum Liquid Chromatography (VLC) and a purification process, the new alkaloid, 11*β*-hydroxygalanthamine (**1**), as well as six known alkaloids, namely galanthamine (**2**), which was found in relatively high quantities, narwedine (**3**), haemanthamine (**4**), 11-hydroxyvittatine (**5**), 8-*O*-demethylmaritidine (**6**) and vittatine (**7**), were isolated and identified by NMR, CD and MS spectrometry (Figure 1). Earlier studies have reported the isolation of galanthamine and other galanthamine-type alkaloids from *Hippeastrum* species, including European cultivars, but mainly as minor compounds.

Compound **1**, 11*β*-hydroxygalanthamine, crystallized as white needles. EI-MS showed a molecular ion peak at *m/z* 303. The base peak at *m/z* 230 evidenced the loss of the hydroxyl group at C-3 and the C-11/C-12/NMe residue, in agreement with other galanthamine-type alkaloids [11]. The ^1^H-NMR of compound **1** (Table 1) was very similar to that of habranthine previously isolated from *Habranthus brachyandrum* and *Pancratium maritimum* [12,13], with small differences in the chemical shifts of H-11 and H-1. The key to the assignation was the large coupling constant *J*_(11*α*,12*β*)_ = 10.8 Hz, observed in compound **1,** which indicates a *trans* diaxial relationship between H-11*α* and H-12*β*, and therefore a *β*-position for the hydroxyl substituent. In contrast, the coupling constants observed for the H-11 of habranthine (*J*_(11*β*,12*β*)_ = 1.6 Hz) and (*J*_(11*β*,12*α*)_ = 4.5 Hz) indicated that the hydroxyl-substituent should be in the *α*-position (endo). Moreover, the H-12 protons in compound **1** are clearly separated in a double doublet, and 2D analysis confirmed the correct assignment, where NOESY correlations between H-12*β* and H-4/H-6*β* and between H-12*α* and NMe were observed. The complete assignment of the 11*β*-hydroxygalanthamine is presented in Table 1.

Alkaloid **1** has also proven to be an inhibitor of acetylcholinesterase like the majority of the galanthamine-type alkaloids. In an acetylcholinesterase inhibition screening of several Amaryllidaceae alkaloids, López *et al*. [14] found that habranthine, epimer of compound **1**, showed similar activity to galanthamine. The *β*-configuration of the hydroxyl group at position 11 in **1** could be unfavourable for its interactions within the active site of the acetylcholinesterase enzyme. The IC_50_ for **1** was 14.5 ± 0.33 µM, while galanthamine showed an IC_50_ of 1.18 ± 0.07 µM.

**Table 1:** ^1^H NMR, COSY, NOESY, HSQC and HMBC data of 11*β*-hydroxygalanthamine (400 MHz, CDCl_3_).

Position	H δ	H δ (J in Hz)	COSY	NOESY	HSQC			HMBC
1	4.88	*br s*	H-2*α*, H-2*β*	H-2*α*, H-2*β*, H-11*α*		89.0	*d*	C-3, C-4a, C-11
2*α*	2.70	*ddd* (15.6, 5.2, 2.8)	H-1, H-2*β*	H-1, H-2*β*, H-3		32.3	*t*	C-1, C-3, C-4, C-10b
2*β*	2.37	*br dt* (15.6, 1.6)	H-1, H-2*α*, H-3	H-1, H-2*α*, H-3				--
3	4.20	*br t* (4.8)	H-2*β*, H-4	H-2*α*, H-2*β*, H-4		62.2	*d*	C-1, C-4, C-4a
4	6.29	*dd* (10.4, 4.8)	H-3, H-4a	H-3, H-4a		133.1	*d*	C-2, C-10b
4a	5.96	*d* (10.0)	H-4	H-4, H-6*β*, H-12*β*		122.6	*d*	C-1, C-3, C-10b
6*α*	3.60	*d* (15.2)	H-6*β*	H-6*β*, H-7, NMe		59.5	*t*	C-6a, C-7, C-10a, C-12, NMe
6*β*	3.93	*d* (14.8)	H-6*α*	H-4a, H-6*α*, H-12*β*				C-6a, C-7, C-10a, C-12, NMe
6a						129.9	*s*	
7	6.61	*d* (8.0)	H-8	H-6*α*, H-8		122.4	*d*	C-6, C-9, C-10a
8	6.68	*d* (8.0)	H-7	H-7, OMe		111.8	*d*	C-6a, C-10
9						144.6	*s*	
10						146.9	*s*	
10a						128.9	*s*	
10b						53.4	*s*	
11*α*	4.05	*dd* (10.8, 4.0)	H-12*α*, H-12*β*	H-1, H-12*α*, H-12*β*, NMe		72.0	*d*	C-1, C-4a
12*α*	3.02	*dd* (14.0, 3.2)	H-11*α*, H-12*β*	H-11*α*, H-12*β*, NMe		62.1	*t*	C-6, C-10b, C-11
12*β*	3.17	*dd* (13.6, 10.8)	H-11*α*, H-12*α*	H-4a, H-6*β*, H-11*α*, H-12*α*				C-6, C-11, NMe
OMe	3.84	*s* (3H)		H-8		56.2	*q*	C-9
NMe	2.45	*s* (3H)		H-6*α*, H-11*α*, H-12*α*		43.2	*q*	C-6, C-12

## 3. Experimental

### 3.1. General

NMR spectra were recovered in a Varian Mercury 400 MHz instrument using CDCl_3_ (CD_3_OD for compound **5**) as a solvent and TMS as the internal standard. Chemical shifts were reported in δ units (ppm) and coupling constants (*J*) in Hz. EIMS were obtained on a GC-MS Hewlett-Packard 6890+ MSD 5975 operating in EI mode at 70 Ev.

An HP-5 MS column (30 m × 0.25 mm × 0.25 µm) was used. The temperature program was: 100–180 °C at 15 °C min^–1^, 1 min hold at 180 °C and 180–300 °C at 5 °C min^–1^ and 10 min hold at 300 °C.Injector temperature was 280 °C. The flow rate of carrier gas (Helium) was 0.8 mL min^–1^. Split ratio was 1:20. A QSTAR Elite hybrid Quadrupole-Time of Flight (QToF) mass spectrometer (Applied Biosystems, PE Sciex, Concord, ON, Canada) was used for HR-MS analysis. ToF MS data were recorded from *m/z* 70 to 700 amu with an accumulation time of 1 s and a pause between the mass range of 0.55 ms, operating in the positive mode. Reserpine (1 ρmol/µL) in product ion scan mode of *m/z* 609 was used for calibration of the mass spectrometer. Optical rotations were carried out on a Perkin-Elmer 241 polarimeter. A Jasco-J-810 Spectrophotometer was used to run CD spectra, all recorded in MeOH. UV spectra were obtained on a DINKO UV2310 instrument and IR spectra were recorded on a Nicolet Avatar 320 FT-IR spectrophotometer.

### 3.2. Plant Material

*Hippeastrum papilio* was collected during the flowering period (November, 2009) in the South of Brazil (Caxias do Sul-RS). A voucher specimen (ICN-149428) has been deposited in the Institute of Botany, Universidade do Rio Grande do Sul (UFRGS), Porto Alegre, and identified by Julie Dutilh PhD, University of Campinas.

### 3.3. Extraction and Isolation of Alkaloids

Fresh bulbs (2 Kg) were crushed and exhaustively extracted with EtOH (96% v/v) at room temperature for 48 h and the combined macerate was filtered and evaporated to dryness under reduced pressure. The bulb crude extract (50 g) was acidified to pH 2 with diluted H_2_SO_4_ and extracted with Et_2_O (4 × 250 mL) to remove neutral material. The aqueous solution was basified with 25% ammonia up to pH 11 and extracted with *n*-hexane (8 × 250 mL) to give extract A (0.55 g). Another extraction using EtOAc (8 × 250 mL) gave extract B (1.2 g) and the last extraction using EtOAc-MeOH (3:1, 3 × 250 mL) gave extract C (3.4 g). Extract A yielded galanthamine (**2**) by crystallization from acetone. Extract B was subjected to a VLC column (3 × 6 cm) using silica gel (250 g – Kieselgel – mesh 0.15/0.30), eluting with *n*-hexane gradually enriched with EtOAc (0 → 100%) and then with MeOH (0→50%). Fractions of 100 mL were collected (190 in total) monitored by TLC (Dragendorff’s reagent, UV light λ 254 nm) and combined according to their TLC profiles, obtaining three fractions: 70–90 (fraction **I**), 100–124 (fraction **II**) and 125 – 145 (fraction **III**). From **I**, galanthamine (**2**, 150 mg, 0.0075% of fresh bulbs) was isolated again by crystallization from acetone. Fraction **II** (250 mg) was subjected to a VLC column (1,5 × 3,5 cm) using *n*-hexane gradually enriched with EtOAc (0–100%) and then with MeOH (0–50%), providing 100 fractions. After combining fractions 55–85, PTLC (20 cm × 20 cm × 0.25 mm, Silica gel F_254_, EtOAc:CHCl_3_:*n*-Hexane:MeOH = 4:2:2:1, v/v/v/v, in NH_3_ atmosphere) was used to isolate haemanthamine (**4**, 80 mg) and 8-*O*-demethyl-maritidine (**6**, 3.5 mg). From **III**, using PTLC (20 cm × 20 cm × 0.25 mm, Silica gel F_254_, EtOAc-CHCl_3_-MeOH = 4:2:1, in NH_3_ atmosphere) 11-hydroxyvittatine (**5**, 10mg) and 11*β*-hydroxy-galanthamine (**1**, 55 mg, 0.00275% of fresh bulbs) were isolated. Fresh leaves (approx. 1Kg) were also submitted to alkaloid extraction. Their alkaloid profile obtained by TLC and GC-MS was quite similar to that observed for bulbs, with additional traces of narwedine (**3**) and vittatine (**7**), which were identified by comparing their GC-EI-MS spectra and Kovats retention indices (RI) with our own library database. All known alkaloids isolated were identified by comparing their physical and spectroscopic data with those of alkaloids previously isolated and characterized by our group [4,15,16,17,18].

### 3.4. Microplate AChE Assay

The assay for measuring AChE activity was performed as described by López *et al*. [14]. Galanthamine hydrobromide was used as a positive control. The IC_50_ of 11*β*-hydroxygalanthamine, galanthamine hydrobromide and galanthamine was measured in triplicate and the results are presented as a mean ± standard deviation using the software package Prism (Graph Pad Inc., San Diego, USA). Both isolated alkaloids and the positive control were evaluated at a concentration ranging from 10^–3^ M to 10^–8^ M.

*11β-Hydroxygalanthamine* (**1**). White needles. UV (MeOH) λ_max_ nm: 212.5, 287. [*α*]^D^_24_ = –20° (*c* 1.1, CHCl_3_); CD [Θ]^20^_λ_: [Θ]_230_ +586, [Θ]_247_ –1950, [Θ]_291_ +3782. IR (CHCl_3_) ν_max_ cm^–1^: 3360, 2925, 2854, 1730, 1624, 1590, 1508, 1440, 1280, 1096, 1044, 977, 756. ^1^H-NMR, COSY, NOESY, HSQC, HMBC (400 MHz, CDCl_3_) and ^13^C-NMR (100 MHz, CDCl_3_) see Table 1. EI-MS 70eV (rel. int.): 303(M^+^, 32), 302(18), 286(11), 231(22), 230(100), 213(25), 181(12), 174(10), 97(96), 57(12). HR-QTOF-MS [M + H]^+^: 304.1550 (cald for C_17_H_22_NO_4_, 304.1549).

## 4. Conclusions

Although the chemical synthesis of galanthamine has been achieved, its supply for clinical use still comes from natural sources. *Hippeastrum papilio* is able to produce great quantities of galanthamine but more studies on its genetic improvement, hybridization or *in vitro* culture are needed. Our search for new sources of galanthamine and acetylcholinesterase inhibitors has resulted in the isolation and identification of 11*β*-hydroxygalanthamine (**1**), a new acetylcholinesterase inhibitor alkaloid. The compound was structurally elucidated by 2D NMR, which allowed us to distinguish it from its epimer, habranthine. Galanthamine-type alkaloids are well-known for their inhibitory activity of the acetylcholinesterase enzyme. The action of galanthamine as an allosterically potentiating ligand in nicotinic acetylcholine receptors [5] and its ability to inhibit *β*-amyloid aggregation [19] could also play a role in successful AD therapy. The discovery of new galanthamine-type candidates is therefore of real interest for the future management of this disease.

## References

[1] Bastida J., Lavilla R., Viladomat F., Cordell G.A. (2006). Chemical and biological aspects of *Narcissus* alkaloids. The Alkaloids.

[2] Sener B., Orhan I., Satayavivad J. (2003). Antimalarial activity screening of some alkaloids and the plant extracts from Amaryllidaceae. Phytother. Res..

[3] McNulty J., Nair J.J., Bastida J., Pandey S., Griffin C. (2009). Structure-activity studies on the lycorine pharmacophore: a potent inducer of apoptosis in human leukemia cells. Phytochemistry.

[4] Berkov S., Codina C., Viladomat F., Bastida J. (2008). *N*-alkylated galanthamine derivatives: potent acetylcholinesterase inhibitors from *Leucojum aestivum*. Bioorg. Med. Chem. Lett..

[5] Maelicke A., Samochocki M., Jostock R., Fehrenbacher A., Ludwig J., Albuquerque E.X., Zerlin M. (2001). Allosteric sensitization of nicotinic receptors by galantamine, a new treatment strategy for Alzheimer’s Disease. Biol. Psychiatry.

[6] Berkov S., Bastida J., Viladomat F., Codina C. (2011). Development and validation of a GC-MS method for rapid determination of galanthamine in *Leucojum aestivum* and *Narcissus* ssp.: A metabolomic approach. Talanta.

[7] da Silva A.F.S., de Andrade J.P., Bevilaqua L.R.M., de Souza M.M., Izquierdo I., Henriques A.T., Zuanazzi J.A.S. (2006). Anxiolytic-, antidepressant- and anticonvulsant-like effects of the alkaloid montanine isolated from *Hippeastrum vittatum*. Pharmacol. Biochem. Behav..

[8] Pagliosa L.B., Monteiro S.C., Silva K.B., de Andrade J.P., Dutilh J., Bastida J., Cammarota M., Zuanazzi J.A.S. (2010). Effect of isoquinoline alkaloids from two *Hippeastrum* species on *in vitro* acetylcholinesterase activity. Phytomedicine.

[9] Giordani R.B., Vieira P.B., Weizenmann M., Rosemberg D.B., Souza A.P., Bonorino C., de Carli G.A., Bogo M.R., Zuanazzi J.A.S., Tasca T. (2010). Candimine-induced cell death of the Amitochondriate Parasite *Trichomonas vaginalis*. J. Nat. Prod..

[10] Meerow A.W., Guy C.L., Li Q.B., Yang S.L. (2000). Phylogeny of the American Amaryllidaceae based on nrDNA ITS sequences. Syst. Bot..

[11] Hesse M., Berhard H.O., von Budzikiewicz H. (1975). Amaryllidaceae alkaloids. Progress in Mass Spectrometry.

[12] Wildman W.C., Brown C.L. (1968). The structure of habranthine. Tetrahedron Lett..

[13] Tato M.P.V., Castedo L., Riguera R. (1988). New alkaloids from *Pancratium maritimum* L. Heterocycles.

[14] López S., Bastida J., Viladomat F., Codina C. (2002). Acetylcholinesterase inhibitory activity of some Amaryllidaceae alkaloids and *Narcissus* extracts. Life Sci..

[15] Bastida J., Viladomat F., Llabrés J.M., Codina C., Feliz M., Rubiralta M. (1987). Alkaloids from *Narcissus confusus*. Phytochemistry.

[16] Bastida J., Contreras J.L., Codina C., Wright C.W., Phillipson J.D. (1995). Alkaloids from *Narcissus cantabricus*. Phytochemistry.

[17] Berkov S., Bastida J., Tsvetkova R., Viladomat F., Codina C. (2009). Alkaloids from *Sternbergia colchiciflora*. Z. Naturforsch. C.

[18] Bastida J., Bergoñón S., Viladomat F., Codina C. (1994). Alkaloids from *Narcissus primigenius*. Planta Med..

[19] Matharu B., Gibson G., Parsons R., Huckerby T.N., Moore S.A., Cooper L.J., Millichamp R., Allsop D., Austen B. (2009). Galantamine inhibits *β*-amyloid aggregation and cytotoxicity. J. Neurol. Sci..

